# Optimization and Technical Validation of the AIDE-MOI Fall Detection Algorithm in a Real-Life Setting with Older Adults

**DOI:** 10.3390/s19061357

**Published:** 2019-03-18

**Authors:** Simon Scheurer, Janina Koch, Martin Kucera, Hȧkon Bryn, Marcel Bärtschi, Tobias Meerstetter, Tobias Nef, Prabitha Urwyler

**Affiliations:** 1 Department of Engineering and Information Technology, Bern University of Applied Sciences, 3401 Burgdorf, Switzerland; simon.scheurer@oxomed.ch (S.S.); janina.koch@oxomed.ch (J.K.); martin.kucera@bfh.ch (M.K.); haakon.bryn@gmail.com (H.B.); marcel.baertschi@gmail.com (M.B.); tm@oxon.ch (T.M.); 2 Oxomed AG, 3097 Liebefeld, Switzerland; 3 Gerontechnology and Rehabilitation Group, University of Bern, 3008 Bern, Switzerland; tobias.nef@artorg.unibe.ch; 4 ARTORG Center for Biomedical Engineering Research, University of Bern, 3008 Bern, Switzerland; 5 University Neurorehabilitation Unit, Department of Neurology, University Hospital Inselspital, 3010 Bern, Switzerland

**Keywords:** wearable, fall detection, healthcare, sensors, threshold algorithm

## Abstract

Falls are the primary contributors of accidents in elderly people. An important factor of fall severity is the amount of time that people lie on the ground. To minimize consequences through a short reaction time, the motion sensor “AIDE-MOI” was developed. “AIDE-MOI” senses acceleration data and analyzes if an event is a fall. The threshold-based fall detection algorithm was developed using motion data of young subjects collected in a lab setup. The aim of this study was to improve and validate the existing fall detection algorithm. In the two-phase study, twenty subjects (age 86.25 ± 6.66 years) with a high risk of fall (Morse > 65 points) were recruited to record motion data in real-time using the AIDE-MOI sensor. The data collected in the first phase (59 days) was used to optimize the existing algorithm. The optimized second-generation algorithm was evaluated in a second phase (66 days). The data collected in the two phases, which recorded 31 real falls, was split-up into one-minute chunks for labelling as “fall” or “non-fall”. The sensitivity and specificity of the threshold-based algorithm improved significantly from 27.3% to 80.0% and 99.9957% (0.43) to 99.9978% (0.17 false alarms per week and subject), respectively.

## 1. Introduction

With a steady growth of the aging population, falls have become a concern to quality of life and health services. From the age of 65 onwards, one in three people fall at least once a year [1], and the risk of falling increases with age [2]. Falls are one of the main causes for accidents and deaths among elderly people [2]. The time people remain on the ground is an influential factor on the severity of the consequences of a fall [3]. To lie on the floor for at least one hour is defined as a “long-lie” [4]. Long lies often lead to physical consequences, such as dehydration, hypothermia, and muscle damage [5,6]. The damage due to a fall can be greatly reduced by providing a means of quick and safe rescue.

Furthermore, psychological aftermaths can also appear. While the fear of falling is the most common, it is often the beginning of a vicious circle; a fear of falling leads to inactivity and lack of movement, which increases the risk of recurrent falls [7]. The combination of psychological and physical consequences often results in dependency on long-term care, social isolation and lower quality of life [3]. About half of the elderly people who have fallen are unable to get up without assistance, even when no injury has occurred [3]. There are several supporting emergency devices, which can be activated after a fall to get help. Nevertheless, only about 20% of fallen people use wearable alarm system(s) to summon help [8]. This calls for further research and development to demonstrate the importance of automatic fall detection systems.

An automatic fall detection system is a system that sends an alert for help when a fall has occurred. These systems consist of a network of one or more sensors, an alarm telephone or watch, and a detection algorithm. Depending on the sensor type, these systems can be classified into two main categories: wearable and non-wearable systems [9]. A systematic review shows that about 60% of identified projects use wearable systems [10]. Wearable systems are attached to the subject’s body and can measure the subject’s motion using one or several kinds of sensors. The most commonly used wearable sensors are: accelerometer (95%), gyroscope (7%), pressure (3%), and a switch (3%) [11,12]. The advantages of wearable systems are their ability to monitor the subject continuously, irrespective of their location [9]. The protection of patient privacy by not using cameras or microphones [9,13] leads to higher acceptance. Non-wearable systems include mostly optical sensors, cameras, motion sensors, microphones, and floor sensors [10,14]. An advantage of non-wearable systems is that they do not have to be worn by the user, leading to higher user compliance [15]. The drawback of non-wearable systems is the limited capture area.

Automatic fall detection systems can be also be classified based on the data storage and data processing types: continuous data approach and event-based approach. In the continuous data approach, falls are directly identified in a stream of sensor data that corresponds to user movement, while the event-based approach is based on classifying events as “fall” or not “fall”. Most of the continuous data approach studies do not store the raw sensor data. Some continuous data approach studies have used wearable systems for processing data and raising alarms [16,17], while another study has combined both wearable and non-wearable devices [18]. 

Fall detection algorithms use traditional threshold-based methods [19,20] or new machine learning algorithms to classify falls and non-falls [21]. The advantages of threshold-based classifiers are their easy implementation, low power budget, and low computational complexity [22,23]. Machine learning algorithms for fall detection either use supervised learning to train the classifier or unsupervised learning. Compared to threshold-based classifiers, machine learning classifiers obtain greater performance [24], but have higher complexity, require more computational power, and have a huge amount of representative training data [25]. Studies using machine learning algorithms have reported 98% accurate fall identification with a support vector machine (SVM) [26], and that the kNN classifier outperforms the Bayesian decision-making (BDM), dynamic time warping (DTW), and artificial neural network (ANN) approaches in fall detection [27].

Falls are alarming, but in comparison to normal activities, they are very rare events. Statistics from the USA report that the average fall rate in elderly care homes is 2.6 per person per year [13]. The rare occurrence of falls in real life is one of the main reasons why a clear majority of studies have used simulated data. Recent systematic reviews have demonstrated that less than 7% of the relevant studies used real-life falls [10,11]. In addition, when collecting data from real-life falls there are data privacy issues [28], as well as some ethical issues that must be considered, since the participants of the study have to undergo a real fall that may be harmful. Nevertheless, it is important to use real-life fall data, as classification models built using artificial falls are often poor representations of actual falls [13,19,29,30], adding to the poor external validity [31] of simulated falls. 

Falls are multifactorial incidents caused by a complex interaction of intrinsic problems, extrinsic factors, and exposures [11]. There is a huge diversity in how and where falls may occur. Falls characteristics may differ based on the direction, velocity, or acceleration of the body or body parts prior to, or during a fall. Falls also show a high intra- and inter-individual variability. A literature review focusing on the type-location of wearable sensors and fall assessment during tasks concluded that no general rule can be deduced from the different methodologies used to date [32], but emphasizes on placing sensors at positions capable of capturing the static and dynamic stability of the user. The heterogeneity of falls illustrates the difficulty to generalize the analysis from one fall to another. According to a systematic review [11], 75% of fall-detection studies are conducted with healthy young participants and thus do not represent the real target population. Data gathered from these studies lack either enough sensitivity or specificity for generalization to other age groups.

In summary, previous studies often highlight issues concerning the generalization of the gained data, either due to use of simulated data or due to the non-involvement of target age group. To overcome these various gaps, the wearable motion sensor “AIDE-MOI” was developed in close collaboration with elderly people [33]. “AIDE-MOI” is a technical assistance system that ensures immediate assistance after a fall, even when elderly people are incapable of summoning assistance with a manual device. The system records acceleration data and analyzes the data for fall occurrences. In case of a fall, the sensor automatically sends out help requests. The first-generation threshold-based fall detection algorithm was developed and validated at the Bern University of Applied Sciences using experimental fall data of young subjects in a laboratory setup. The algorithm was based on the three features: upper peak value (UPV), max convolution (CONV), and variance after fall (VAF). The aim of this study was to improve and validate the first-generation fall detection algorithm using real-life data from elderly people with a high fall risk.

## 2. Materials and Methods

### 2.1. Material–Sensor System

**Figure 1:**
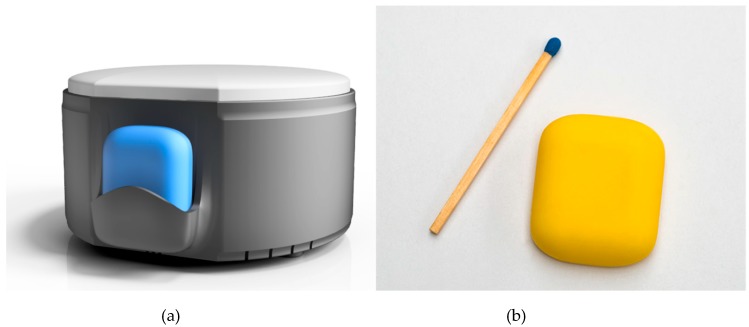
The system “AIDE-MOI” consists of a home station (**a**) and a fall detection sensor (**b**).

**Figure 2:**
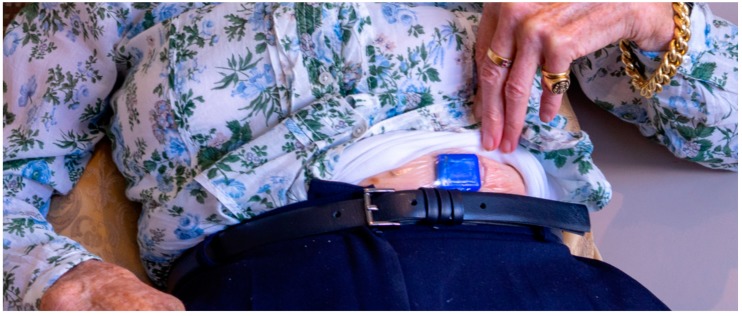
The sensor is attached to the participant’s body with an adhesive plaster. The sensor is worn under the clothes and hence not visible to others.

The AIDE-MOI system consists of a home station and a fall detection wearable sensor, as shown in Figure 1. The wearable sensor is the main part of the system. The sensor is small (*l* × *b* × *h* 25 × 30 × 8 mm, weight 7.2 g) enough to be easily wearable as stated in a recent review on medical sensors in Internet of Things [34]. As shown in Figure 2, the sensor can be attached to the body and records the acceleration (3 axis accelerometer [35], 100 Hz, +/− 8 g, 10 bit resolution) and temperature (+/− 2 °C) of the user. In case of a fall, the sensor sends an alarm to the nearest home station via a 2.4 GHz long range radio (EM9209) [36]. Due to the proprietary radio protocol (LoRa^TM^) and the low baud rate, data can be transmitted over several hundred meters. LoRa^TM^ is a physical wireless protocol based on a unique modulation format (frequency modulated (FM) chirp) allowing for connections in a range of many kilometers. The electronics are covered with a biocompatible silicone coating to protect against irritation and skin reactions. The complete sealing makes the sensor waterproof, and it can be worn during showering and bathing. The sensor is equipped with 1 Gbit of memory [37], which enables it to store motion data and system information for more than two and a half weeks. The recorded data can be downloaded via Bluetooth. The sensor can easily be recharged wirelessly using the home station (Qi-Standard). The optimal resolution, sampling frequency and other sensor characteristics were analyzed and studied in a previous study [33].

### 2.2. Subject Recruitment

The data collection was carried out in accordance with the latest version of the Declaration of Helsinki and was approved by the Ethics Committee of the Canton of Bern, Switzerland. All data collection and data storage methods followed the requirements of the data protection law to encounter the challenges of security, data protection, and privacy [28]. Personal data were anonymized and stored on a secure HIPAA (Health Insurance Portability and Accountability Act of 1996) compliant web-based electronic data capture system. Access to the data was only given to authorized users. All procedures related to the study were explained to the participants, and a written informed consent was obtained prior to participation. Only subjects (or their legal representatives in case of persons incapable of judgment) who were able to give a written informed consent were included in the study. No compensation for participation was provided. Elderly people (>65 years) were recruited via advertisement (a website) and through care homes. Demographic details were collected using standard questionnaires. Subjects were assessed for neuropsychological details with standardized paper–pencil test battery, which included the Montreal Cognitive Assessment (MoCA) [38] for global cognition, the Morse Fall Scale (MFS) [39,40] for assessment of fall risk and the Katz-Index test [41,42] to measure independence of activities during daily living. The inclusion criterion was high risk of fall (>55 points), as indicated in the MFS. Subjects with known plaster allergies were excluded from the study. 

A total of twenty elderly participants (age 86.25 ± 6.66 years) were included in the study. From the total pool of twenty participants, eleven elderly people participated in the first phase and eighteen elderly people participated in the second phase. Eleven participants (55%) had a clinical diagnosis of dementia. All subjects were registered under a “need for care” service and had assistance at least once a day, except for one subject, who was rendered care one to six times per week. Different types of walking aids (walking stick (5%), walkers (30%), rollator (55%)) were used by the participants for mobility. 

### 2.3. Study

The study was split into two phases, and the total study spanned over a period of four months. Each participant could participate for a maximum of two months. The first phase had a duration of 59 days and the second phase had a duration of 66 days. From the point of view of the subjects, both phases were identical, as the hardware (sensor) was the same for both phases. 

In the first phase, the sensor was loaded with the first-generation algorithm, allowing it to be validated with real-life data of elderly subjects. In addition, the data collected during this phase was used to improve the algorithm for the second phase. The improved, second-generation algorithm was validated in the second phase. 

### 2.4. Sensor Data Collection 

After baseline assessments, the regular sleeping position was discussed to select a suitable attachment position for the sensor. The sensor was attached either to the back, abdomen, or chest with a plaster. The orientation of the axis did not hinder detection performance for sensor locations in the torso region [32]. In addition, the home stations were placed in the subjects’ rooms and in places where they spent a significant amount of time. To guarantee a comprehensive analysis, the participants and care professionals were requested to record a project event protocol in case of a fall or a false alarm. The fall detection sensor was officially exchanged by the researcher once a week. A new sensor was attached to a location adjacent to the original. The detached sensor was taken back to the laboratory for data download via Bluetooth. On completion of the data transfer, the sensor memory was cleared, fully recharged, cleaned with disinfectant and sealed in a small plastic bag for the next user. 

### 2.5. Fall Definition

A fall is defined as “an unexpected event in which the participants come to rest on the ground, floor, or lower level” [43], while Yang et al. [44] specified a fall as an event that occurs in a period shorter than one second. Usually a fall event takes between 0.45 and 0.85 s [44]. During a fall, the posture and shape of the person changes significantly [44].

A fall is split up into three phases: pre-fall, impact, and post-fall [11]. The pre-fall phase is the period in which the person is falling. In this phase, the acceleration towards the earth is smaller than 1g. The impact phase, which is the moment when the person hits the ground, plays an important role in the fall detection algorithm. Often, the impact phase is taken as a reference point in algorithms, because it is easy to detect. The post-fall phase describes the time after the person hits the ground. In this phase, the fallen person is either lying down, sitting still, or trying to stand up. 

### 2.6. Feature Selection

The raw data were split up into one-minute slices (6000 samples). Each slice was labeled as “fall” or “non-fall”. Using statistical models, features were calculated from the raw data of the accelerometer and temperature sensors. Most of the features were based either on the raw tri-axial acceleration data (a_x_, a_y_, a_z_) or on the Root-Sum-of-Squares (RSS_ACC_) of the tri-axial accelerometer signal:

(1) $$RSS_{ACC}=\sqrt{a_{x}{\left(t\right)}^{2}+a_{y}{\left(t\right)}^{2}+a_{z}{\left(t\right)}^{2}}-1g$$

The classification of the three phases (pre-fall, impact, and post-fall) is important to achieve high sensitivity [45]. Therefore, most of the features described below take all the three phases into account. 

**Figure 3:**
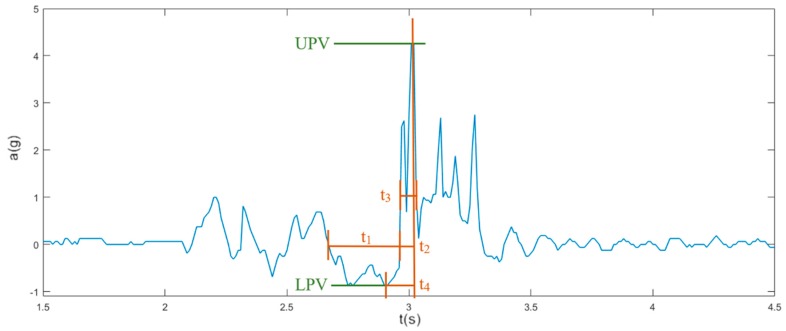
Illustration of the fall phases with their time representations: duration of the pre-fall phase *t*_1_, duration of the main peak t_2_, width of the main peak *t*_3_, and t_4_ is the time difference between lower peak value (LPV) and upper peak value (UPV).

Figure 3 shows the normalized acceleration *a* of a real fall depending on the normalized time *t* in seconds. The upper peak value (UPV) is calculated using the RSS_ACC_. UPV is one of the most important features and is taken as a reference point for time. The lower peak value (LPV) is defined as the lowest RSS_ACC_ value in a window of 500 ms before the UPV. Four different times, *t*_1_, *t*_2_, *t*_3_ and *t*_4_, as shown in Figure 3, were calculated for each slice. These mainly describe the pre-fall phase and the impact phase. In the pre-fall phase (t_1_), the acceleration towards the earth is smaller than 1g. Time *t*_1_ ranges in a window of 500 ms before the UPV, when the RSS_ACC_ varies between −0.125 g and 0.125 g. The rising time of the main peak is described by *t*_2_, starts with a window of 200 ms before the UPV when the RSS_ACC_ rises above 0.125 g, and ends at the UPV. The duration of the peak is denoted by *t*_3_, in which the UPV is present. The window starts when the RSS_ACC_ rises above ¼ of the UPV and ends when the RSS_ACC_ drops below ¼ of the UPV. The time difference between LPV and UPV is *t*_4_. 

To calculate max convolution (CONV)_RSS_, the RSS_ACC_ was compared to a normal fall, which was determined by more than 100 simulated falls of young subjects from the first-generation “AIDE-MOI” study. The comparison of simulated norm falls with the measured RSS_ACC_ followed a convolution (Equation (2)), where *h* represents the norm fall with the length *p* and *x(n)* the incoming RSS_ACC_ signal. For the feature selection, the maximal convolution value was calculated from each slice (Equation (3)):

(2) $$r_{xh}\left(n\right)=\sum_{i=0}^{p-1}h\left(i\right)*x\left(n-i\right)$$

(3) $$CONV_{RSS}=\max\left(r_{xh}\left(n\right)\right)$$

**Figure 4:**
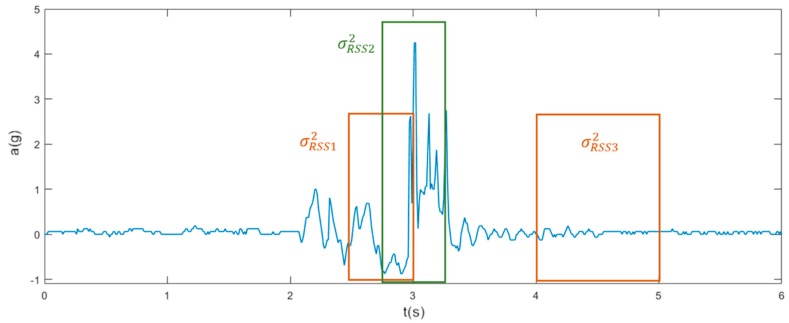
Time window (boxes) illustration for variance calculation of each phase: variance of the pre-fall phase (σ^2^_RSS1_), impact phase (σ^2^_RSS2_), and post-fall phase (σ^2^_RSS3_).

As shown in Figure 4, the variance of RSS_ACC_ was calculated using Equation (4) for each phase of a fall, where µ_RSS_ is the mean of the RSS_ACC_ values and N_RSS_ is the number of RSS_ACC_ values. The variance of the pre-fall phase (σ^2^_RSS1_) is calculated for 400 ms before the UPV to the UPV and for the impact phase (σ^2^_RSS2_) from 200 ms prior to UPV to 200 ms after the UPV. The variance of the post-fall phase is (σ^2^_RSS3_) calculated in the time window, which ranges from one to two seconds after the UPV:

(4) $$\sigma_{RSS}^{2}=\frac{\sum {\left(RSS-\mu_{RSS}\right)}^{2}}{N_{RSS}}$$

The variance of acceleration (σ^2^_acc_) was calculated for each axis and all three phases. The time window and positions are the same as for the variance calculation of RSS_ACC_. The variances were calculated with the Equations (5) and (6):

(5) $$\sigma_{acc_{x}}^{2}=\frac{\sum {\left(acc_{x}-\mu_{acc_{x}}\right)}^{2}}{N_{acc_{x}}}$$

(6) $$\sigma_{acc}^{2}=\sqrt{\sigma_{acc_{x}}^{2}{}^{2}+\sigma_{acc_{y}}^{2}{}^{2}+\sigma_{acc_{z}}^{2}{}^{2}}$$

**Figure 5:**
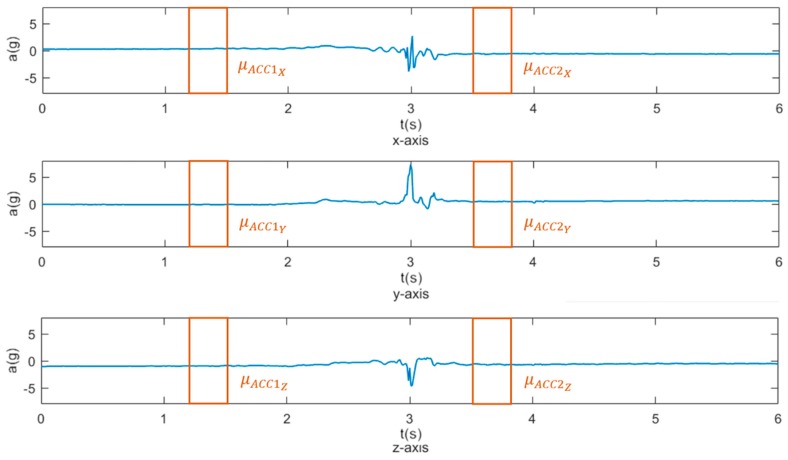
Posture change is detected by measuring acceleration differences for each axis. The boxes represent the 300 ms long window for calculation of mean acceleration before (µ_ACC1_) and after (µ_ACC2_) the upper peak value.

To capture a person’s change of posture during a fall event, mean values of each axis were calculated 1.8 seconds before (µ_ACC1_) and 0.5 seconds after the UPV (µ_ACC2_), as shown in Figure 5. Each window was 300 ms long. The mean difference of each axis was calculated (Equation (7)), and the RSS of the mean difference was taken from all axes (Equation (8)):

(7) $$\Delta \mu_{ACC_{X}}=\mu_{ACC1_{X}}-\mu_{ACC2_{X}}$$

(8) $$\Delta \mu_{ACC}=\sqrt{\Delta \mu_{ACC_{X}}{}^{2}+\Delta \mu_{ACC_{Y}}{}^{2}+\Delta \mu_{ACC_{Z}}{}^{2}}$$

**Figure 6:**
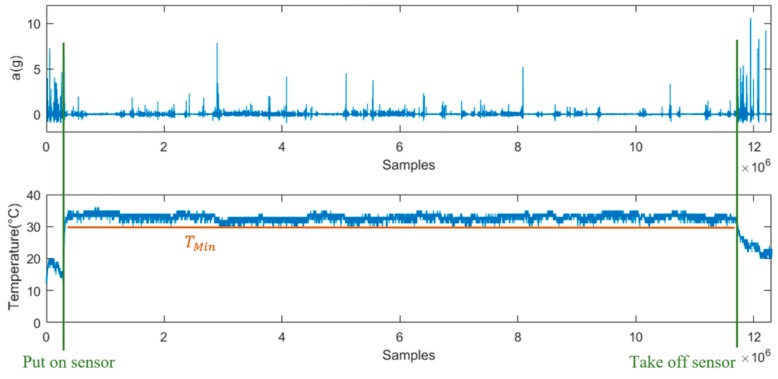
Figure depicting the acceleration Root-Sum-of-Squares (RSS_ACC_) (top) and temperature (below) values over one week, starting with a rise in temperature indicating sensor attachment, constant temperature (*T_min_*) when the sensor being worn, drop in temperature when sensor is detached.

The temperature was measured to detect if the sensor was worn or not. The minimum temperature (T_min_) value was calculated during the wearing period (Figure 6).

The two features, UPV and CONV_RSS_, were already implemented in the first-generation algorithm. The second-generation algorithm is based on 15 different features: UPV, LPV, CONV_RSS_, *t*_1_, *t*_2_, *t*_3_, *t*_4_, σ^2^_RSS1_, σ^2^_RSS2_, σ^2^_RSS3_, σ^2^_ACC1_, σ^2^_ACC2_, σ^2^_ACC3_ $\Delta \mu_{ACC}$ and *T_min_.*

### 2.7. Analysis

The aim of this analysis was to detect features which may make a significant contribution to the differentiation between falls and non-falls. The Mann-Whitney-U-Test was used to detect significant feature differences for classifying events as falls and non-falls.

Owing to the limited availability of computational power, energy, and memory on the microcontroller (8 kB random access memory (RAM) and 50 kB flash memory), and due to the unequal size of classes, a simple threshold-based classifier was chosen. The classifier has an upper and lower threshold for each feature. If the feature values were not between these thresholds, the event was classified as a non-fall. A fall was detected if all feature values were within the threshold limits. The thresholds were set based on the training data and to provide maximum sensitivity [16,46,47]. The lower and the upper threshold were set to the highest and the lowest feature values of all training fall data. 

The chi square test of independence was used to compare the metrics of the two algorithms. As the first-generation algorithm was developed using data from young subjects, we gauged the relationship between the age of the participants and the algorithm performance using parametric correlations. 

## 3. Results

### 3.1. Demographics and Data Collection

Table 1 shows the demographic characteristics of each study phase. The average MoCA score of participants with officially diagnosed dementia was 1.2 ± 1.75 (Min: 0, Max 5). Other participants (without known diagnosis of dementia) had an average MoCA score of 16.2 ± 7.33 (Min: 7, Max: 25). 

### 3.2. Features Extraction

The aim of this analysis was to detect features that may make a significant (*p* < 0.05) contribution to the differentiation between falls and non-falls. With the Mann-Whitney-U-Test, the following features were calculated as significant: UPV, LPV, CONV_RSS_, *t*_1_, *t*_2_, σ^2^_RSS1_, σ^2^_RSS2_, σ^2^_ACC1_, σ^2^_ACC2_, and $\Delta \mu_{ACC}$. Based on the significance levels, these features were used to develop the algorithm, except for CONV_RSS_, due to its need of a high computational power and small improvement of the algorithm. The temperature feature was used to turn off the algorithm when the sensor was not worn (T <= 27 °C). 

### 3.3. Algorithms Comparisons

During the entire study, data were recorded over a period of 140 weeks, which corresponds to 1.4 million one-minute slices. In total, 41 falls were documented in the event protocol, of which 31 falls were recorded by the sensor system. The differences in sensitivity, specificity, accuracy, precision and F-measure between the two algorithms are show in Table 2. The second-generation algorithm, with its additional features, performed better than the first-generation algorithm. As shown in Table 2, three out of eleven falls were detected correctly by the algorithm in the first trial phase and sixteen out of twenty falls in the second phase, corresponding to a sensitivity of 27.3% and 80%, respectively. The sensitivity of the second-generation algorithm increased to 100%, while considering only falls with consequences. The 29 false alarms with the first-generation algorithm was reduced to only 10 with the second-generation algorithm, thereby increasing the specificity of the first phase from 99.9957% to 99.9984% for the second phase. This corresponds to 0.43 false alarms per week and 0.17 false alarms per week and subject. In summary, the sensitivity (*p* = 0.006) and specificity (*p* < 0.003) of the new algorithm was significantly higher than the sensitivity and specificity of the first-generation algorithm. Correlation analysis demonstrated a significant negative correlation of age on the false negative values of both the first generation (pearson’s *r* = −0.716, *n* = 9, *p* = 0.030) and second-generation algorithm (pearson’s *r* = −0.767, *n* = 9, *p* = 0.016).

## 4. Discussion

In this paper, we presented the technical validation of the AIDE-MOI fall detection algorithm. The AIDE-MOI system consists of only one sensor, thus keeping the number of sensors to a minimum, which is in line with guidelines followed by other recent projects (e.g., Fall-Mobile Guard) [48]. The wearable sensor can be attached to a comfortable position in the trunk region, as per recommendations of a review on the position of sensors [32]. Moreover, placement on the trunk region also maximizes information on upright posture and dynamics of the user. 

The system has been tested with 20 participants in two phases. The first-generation algorithm was improved by using real motion data from 11 elderly people who participated in the first phase of the study. The second-generation algorithm reached a sensitivity of 80% and a specificity of 99.9985%. These increased metrics are within the sensitivity ranges (62%–100%) and specificity range (87% and 100%) reported for similar studies [18,49,50,51]. The sensitivity of the second-generation further increased to 100%, if falls with consequences are considered. This can be justified by the fact that falls with consequences have a higher UPV because of their stronger impacts, which make them easier to identify. Detecting falls with consequences is an important aspect of a fall detection system. For falls with consequences, help should be quickly rendered, because an influencing factor of the severity of consequences is the time period during which people lie on the ground [8]. In our study, nine participants were injured due to falls. In these cases, an alert was essential for quick first aid. One participant fell because of an epileptic attack. According to the nursing staff, the attack would not have been discovered without the alarm. The epileptic attack occurred at night shortly after the nurses had visited the participant and had returned to other scheduled duties. The next scheduled visit of the participant was hours later, and the epileptic attack would have gone unnoticed or received delayed help.

According to the sociodemographic survey at recruitment, participants fell 1.39 times per month on average, before inclusion in the study. During the study, a reduction in falls was observed (from 1.39 to 1.25 times per month). Reasons for lesser falls may be the sensitization of the participants, relatives and nurses towards fall risks. Another reason may be the immobility of the participants’ due to deteriorating health conditions. 

The false alarms documented in event protocol reduced from 29 in the first phase to 12 in the second phase. The second-generation algorithm was able to reduce the false alarm rate by implementing an additional feature that detects whether the sensor is worn and internally hindering false alarms while the sensor is not being worn. The temperature data (Figure 6) recorded by the fall detection sensor shows that the temperature maintains a level (above 27 °C) and remains constant while the sensor is attached to the body. The drift in temperature due to weather conditions can be justified by the fact that the sensor was worn under the clothes. The threshold value for temperature needs to be validated for different weather conditions. There is a possibility that the ambient temperature in summer can exceed the currently set threshold value of 27 °C, and the activation of the sensor in such cases cannot be ruled out. However, since the sensors are stored in rooms when not in use, the chance that the sensor will turn on while not being worn is not very high. As a further update, the temperature value can be combined with the acceleration value of all three axes to overcome this.

Although the false alarm rate could be reduced from 0.43 false alarms per week to 0.17 false alarms per week and subject, they are still too many false alarms to justify the sensor usage in private homes. This study showed that such an error rate in an elderly care home is justifiable, since checking the alarm takes hardly any time with 24 h assistance available. On the contrary, in a private home, setting off a false alarm means that a family member must travel far to check the alarm, which would involve time and resources for every false alarm. Although false alarms are not a significant problem in elderly care homes, they should be reduced, as every false alarm demands staff visits, thus disturbing the users and their privacy.

The great diversity of fall detection systems reflects the complexity of falls [33]. Our study also showed that the amount of false alarms is highly dependent on individual dynamics, gait, and posture [32]. There were no false alarms for some participants, implying that these participants had a different movement pattern. Alternatively, the algorithm can be made person—specific by training individual data, leading to an increase in sensitivity and specificity of the algorithm. 

One limitation of the present study is the number of participants. Due to the small sample size of twenty participants, the above statements may not be generalized. In addition, a bias cannot be ruled out, as some subjects (*n* = 9) participated in both the first and second phase. Though the participants were recruited individually at different timepoints, the two phases were conducted directly one after the other. Another limitation could be the dementia diagnosis of the participants and the usage of walkers. Participants with dementia function at a very low cognitive level, which may confound the results obtained here. The results obtained with people using walkers cannot be generalized to the bigger target population. Among the twenty participants, nineteen of them were living in eight different long-term care institutions, while one subject was living alone at home. Thus, there may be some factors inherent in the care home environment that influenced the outcomes of this study.

There are several commercial providers for home alerts with fall detection [52]. The AutoAlert fall detection system from Philips Lifeline is one of the leading providers in the USA. In a future study, it would be advisable to compare the services provided by these commercial devices and their performance to the sensor of interest. The multi-phase analysis of the fall events can be extended to posture recognition during the three phases [48]. This can provide enhanced features, such as fall severity, as well as improve the sensitivity. Future improvements to the algorithm should also include detection of postural changes, such as a gradual slide from a seated position, either from a chair or a wheel chair. In addition, the algorithm can be validated for a variety of fall classifications, such as front lying, back lying, etc., to develop performance indexes for each fall classification.

## 5. Conclusions

Reliable detection of falls is crucial to minimizing the consequences of falls. A wide spectrum of data, including rare movement data, can be collected by conducting real-time studies with nursing home residents and single dwelling homes. The data collected from the first phase of the study significantly improved the first-generation threshold-based algorithm of the “AIDE-MOI” system leading to a new second-generation algorithm. 

## Figures and Tables

**Table 1:** Subject demographics (N = 20).

Demographics	First Phase	Second Phase
Number of participants	11 *	18 *
Gender (m/f) (% female)	4/7 (64)	6/12 (67)
Age (years) (mean ± SD)	85.64 ± 7.81	87.50 ± 5.65
Falls history 6 months before study (mean ± SD)	11.45 ± 11.1	7.28 ± 8.87
MoCA score (mean ± SD)	10.64 ± 9.34	6.35 ± 7.52
Katz score (mean ± SD)	1.91 ± 1.38	2.11 ± 1.88
Morse Fall Scale score (mean ± SD)	80.45 ± 8.50	79.44 ± 9.22

* Nine participants participated in both phases. The total participation was limited to two months. MoCA: Montreal Cognitive Assessment.

**Table 2:** Comparison of first- and second-generation algorithm.

	First Phase	Second Phase
Study duration in days	59	66
Amount of data collected (one-minute slices)	675,390	735,872
Number of features used in algorithm	3	15
Real falls documented in event protocol	18	23
Sensor recorded falls	11	20
False alarms documented in event protocol	29	12
True Positive (correctly classified falls)	3	16
True Negative (correctly classified non-falls)	675,350	735,840
False Positive (wrongly classified non-falls)	29	12
False Negative (wrongly classified falls)	8	4
Sensitivity	27.273%	80.0%
Specificity	99.995%	99.998%
Accuracy	99.994%	99.997%
Precision	9.375%	57.143%
F-measure	13.953%	66.666%

## References

[1] Lord S.R., Sherrington C., Menz H.B., Close J.C.T. (2007). Falls in Older People: Risk Factors and Strategies for Prevention.

[2] Fuller G.F. (2000). Falls in the elderly. Am. Fam. Phys..

[3] Tinetti M.E., Liu W.L., Claus E.B. (1993). Predictors and prognosis of inability to get up after falls among elderly persons. JAMA.

[4] Simpson P.M., Bendall J.C., Tiedemann A., Lord S.R., Close J.C. (2014). Epidemiology of emergency medical service responses to older people who have fallen: A prospective cohort study. Prehosp. Emerg. Care.

[5] King M.B., Tinetti M.E. (1995). Falls in community-dwelling older persons. J. Am. Geriatr. Soc..

[6] Mallinson W.J., Green M.F. (1985). Covert muscle injury in aged patients admitted to hospital following falls. Age Ageing.

[7] Roush R.E., Teasdale T.A., Murphy J.N., Kirk M.S. (1995). Impact of a personal emergency response system on hospital utilization by community-residing elders. South Med. J..

[8] Fleming J., Brayne C. (2008). Inability to get up after falling, subsequent time on floor, and summoning help: Prospective cohort study in people over 90. BMJ.

[9] Vallabh P., Malekian R. (2017). Fall detection monitoring systems: A comprehensive review. J. Ambient Intell. Humaniz. Comput..

[10] Chaudhuri S., Thompson H., Demiris G. (2014). Fall Detection Devices and their Use with Older Adults: A Systematic Review. J. Geriatr. Phys. Tehr..

[11] Schwickert L., Becker C., Lindemann U., Maréchal C., Bourke A., Chiari L. (2013). Sturzerkennung mit am Körper getragenen Sensoren: Ein systematischer Review. Z. Gerontol. Geriatr..

[12] Bagala F., Becker C., Cappello A., Chiari L., Aminian K., Hausdorff J.M., Zijlstra W., Klenk J. (2012). Evaluation of accelerometer-based fall detection algorithms on real-world falls. PLoS ONE.

[13] Khan S.S., Hoey J. (2017). Review of fall detection techniques: A data availability perspective. Med. Eng. Phys..

[14] Skubic M., Harris B.H., Stone E., Ho K.C., Bo-Yu S., Rantz M. Testing non-wearable fall detection methods in the homes of older adults. Proceedings of the 2016 38th Annual International Conference of the IEEE Engineering in Medicine and Biology Society (EMBC).

[15] Bosch-Jorge M., Sánchez-Salmerón A.J., Valera Á., Ricolfe-Viala C. (2014). Fall detection based on the gravity vector using a wide-angle camera. Expert Syst. Appl..

[16] Kangas M., Korpelainen R., Vikman I., Nyberg L., Jamsa T. (2015). Sensitivity and false alarm rate of a fall sensor in long-term fall detection in the elderly. Gerontology.

[17] Lipsitz L.A., Tchalla A.E., Iloputaife I., Gagnon M., Dole K., Su Z.Z., Klickstein L. (2016). Evaluation of an Automated Falls Detection Device in Nursing Home Residents. J. Am. Geriatr. Soc..

[18] Bloch F., Gautier V., Noury N., Lundy J.E., Poujaud J., Claessens Y.E., Rigaud A.S. (2011). Evaluation under real-life conditions of a stand-alone fall detector for the elderly subjects. Ann. Phys. Rehabil. Med..

[19] Bourke A.K., O’Brien J.V., Lyons G.M. (2007). Evaluation of a threshold-based tri-axial accelerometer fall detection algorithm. Gait Posture.

[20] Kangas M., Konttila A., Winblad I., Jamsa T. (2007). Determination of simple thresholds for accelerometry-based parameters for fall detection. Conf. Proc. IEEE Eng. Med. Biol. Soc..

[21] Noury N., Fleury A., Rumeau P., Bourke A.K., Laighin G.O., Rialle V., Lundy J.E. Fall detection–principles and methods. Proceedings of the 2007 29th Annual International Conference of the IEEE Engineering in Medicine and Biology Society.

[22] Andò B., Baglio S., Lombardo C.O., Marletta V. (2015). An Event Polarized Paradigm for ADL Detection in AAL Context. IEEE Trans. Instrum. Meas..

[23] Luque R., Casilari E., Morón M.J., Redondo G. (2014). Comparison and characterization of android-based fall detection systems. Sensors.

[24] Gibson R.M., Amira A., Ramzan N., Casaseca-De-La-Higuera P., Pervez Z. (2016). Multiple comparator classifier framework for accelerometer-based fall detection and diagnostic. Appl. Soft Comput. J..

[25] Jin X., Shao J., Zhang X., An W., Malekian R. (2016). Modeling of nonlinear system based on deep learning framework. Nonlinear Dyn..

[26] Albert M.V., Kording K., Herrmann M., Jayaraman A. (2012). Fall classification by machine learning using mobile phones. PLoS ONE.

[27] Ozdemir A.T., Barshan B. (2014). Detecting falls with wearable sensors using machine learning techniques. Sensors.

[28] Guadagni F., Scarpato N., Patrizia F., D’Ottavi G., Boavida F., Roselli M., Garrisi G. (2016). Personal and Sensitive Data in the e-Health-IoT Universe. Lecture Notes of the Institute for Computer Sciences, Social Informatics and Telecommunications Engineering.

[29] Kangas M., Vikman I., Nyberg L., Korpelainen R., Lindblom J., Jämsä T. (2012). Comparison of real-life accidental falls in older people with experimental falls in middle-aged test subjects. Gait Posture.

[30] Klenk J., Becker C., Lieken F., Nicolai S., Maetzler W., Alt W., Zijlstra W., Hausdorff J.M., van Lummel R.C., Chiari L. (2011). Comparison of acceleration signals of simulated and real-world backward falls. Med. Eng. Phys..

[31] Broadley R.W., Klenk J., Thies S.B., Kenney L.P.J., Granat M.H. (2018). Methods for the Real-World Evaluation of Fall Detection Technology: A Scoping Review. Sensors.

[32] Rucco R., Sorriso A., Liparoti M., Ferraioli G., Sorrentino P., Ambrosanio M., Baselice F. (2018). Type and Location of Wearable Sensors for Monitoring Falls during Static and Dynamic Tasks in Healthy Elderly: A Review. Sensors.

[33] Thilo F.J., Bilger S., Halfens R.J., Schols J.M., Hahn S. (2017). Involvement of the end user: Exploration of older people’s needs and preferences for a wearable fall detection device–A qualitative descriptive study. Patient Prefer. Adherence.

[34] Scarpato N., Pieroni A., Di Nunzio L., Fallucchi F. (2017). E-health-IoT universe: A review. Int. J. Adv. Sci. Eng. Inf. Technol..

[35] ST (2008). LIS2DS12–MEMS Digital Output Motion Sensor: Ultra-Low-Power High-Performance 3-Axis “Pico” Accelerometer.

[36] Microelectronic E.M., Sa M. (2014). EM9209: High Sensitivity, 1.5–72 kbps, 2.4 GHz FSK Transceiver.

[37] Micron (2007). NAND Flash Memory: MT29F1G01ABBFDSF.

[38] Nasreddine Z.S., Phillips N.A., Bédirian V., Charbonneau S., Whitehead V., Collin I., Cummings J.L., Chertkow H. (2005). The Montreal Cognitive Assessment, MoCA: A Brief Screening Tool For Mild Cognitive Impairment. J. Am. Geriatr. Soc..

[39] O’Connell B., Myers H. (2002). The sensitivity and specificity of the Morse Fall Scale in an acute care setting. J. Clin. Nurs..

[40] Schwendimann R., De Geest S., Milisen K. (2006). Evaluation of the Morse Fall Scale in hospitalised patients. Age Ageing.

[41] Katz S., Downs T.D., Cash H.R., Grotz R.C. (1970). Progress in development of the index of ADL. Gerontologist.

[42] Katz S., Ford A.B., Moskowitz R.W., Jackson B.A., Jaffe M.W. (1963). Studies of Illness in the Aged. The Index of Adl: A Standardized Measure of Biological and Psychosocial Function. JAMA.

[43] Lamb S.E., Jørstad-Stein E.C., Hauer K., Becker C. (2005). Development of a common outcome data set for fall injury prevention trials: The Prevention of Falls Network Europe consensus. J. Am. Geriatr. Soc..

[44] Yang L., Ren Y., Zhang W. (2016). 3D depth image analysis for indoor fall detection of elderly people. Digit. Commun. Netw..

[45] Hsieh C.Y., Liu K.C., Huang C.N., Chu W.C., Chan C.T. (2017). Novel Hierarchical Fall Detection Algorithm Using a Multiphase Fall Model. Sensors.

[46] Bourke A.K., Lyons G.M. (2008). A threshold-based fall-detection algorithm using a bi-axial gyroscope sensor. Med. Eng. Phys..

[47] Lee J.K., Robinovitch S.N., Park E.J. (2015). Inertial sensing-based pre-impact detection of falls involving near-fall scenarios. IEEE Trans. Neural Syst. Rehabil. Eng..

[48] Fortino G., Gravina R. Fall-MobileGuard: A smart real-time fall detection system. Proceedings of the BodyNets ‘15 the 10th EAI International Conference on Body Area Networks.

[49] Bourke A.K., Klenk J., Schwickert L., Aminian K., Ihlen E.A.F., Mellone S. Fall detection algorithms for real–world falls harvested from lumbar sensors in the elderly population: A machine learning approach. Proceedings of the 2016 38th Annual International Conference of the IEEE Engineering in Medicine and Biology Society (EMBC).

[50] Boyle J., Karunanithi M. Simulated fall detection via accelerometers. Proceedings of the 2008 30th Annual International Conference of the IEEE Engineering in Medicine and Biology Society.

[51] Naranjo-Hernández D., Roa L.M., Reina-Tosina J., Estudillo-Valderrama M.A. (2012). Personalization and adaptation to the medium and context in a fall detection system. IEEE Trans. Inf. Technol. Biomed..

[52] Preece J. Best Fall Detection Sensors of 2019. https://www.toptenreviews.com/health/senior-care/best-fall-detection-sensors/.

